# ABC2A: A Straightforward and Fast Method for the Accurate Backmapping of RNA Coarse-Grained Models to All-Atom Structures

**DOI:** 10.3390/molecules29061244

**Published:** 2024-03-11

**Authors:** Ya-Zhou Shi, Hao Wu, Sha-Sha Li, Hui-Zhen Li, Ben-Gong Zhang, Ya-Lan Tan

**Affiliations:** 1 Research Center of Nonlinear Science, School of Mathematical & Physical Sciences, Wuhan Textile University, Wuhan 430200, China; yzshi@wtu.edu.cn (Y.-Z.S.); 2115173008@mail.wtu.edu.cn (H.W.); 2315173010@mail.wtu.edu.cn (S.-S.L.); hzli@wtu.edu.cn (H.-Z.L.); 2 School of Bioengineering and Health, Wuhan Textile University, Wuhan 430200, China

**Keywords:** RNA 3D structure, coarse-grained model, full atomic structure reconstruction

## Abstract

RNAs play crucial roles in various essential biological functions, including catalysis and gene regulation. Despite the widespread use of coarse-grained (CG) models/simulations to study RNA 3D structures and dynamics, their direct application is challenging due to the lack of atomic detail. Therefore, the reconstruction of full atomic structures is desirable. In this study, we introduced a straightforward method called ABC2A for reconstructing all-atom structures from RNA CG models. ABC2A utilizes diverse nucleotide fragments from known structures to assemble full atomic structures based on the CG atoms. The diversification of assembly fragments beyond standard A-form ones, commonly used in other programs, combined with a highly simplified structure refinement process, ensures that ABC2A achieves both high accuracy and rapid speed. Tests on a recent large dataset of 361 RNA experimental structures (30–692 nt) indicate that ABC2A can reconstruct full atomic structures from three-bead CG models with a mean RMSD of ~0.34 Å from experimental structures and an average runtime of ~0.5 s (maximum runtime < 2.5 s). Compared to the state-of-the-art Arena, ABC2A achieves a ~25% improvement in accuracy and is five times faster in speed.

## 1. Introduction

RNAs play diverse biological roles in living organisms, such as protein synthesis, RNA splicing, and transcription regulation, and the involvement in various human diseases underscores their significance in biological processes [1,2,3]. Moreover, RNAs also have the potential to be used as therapeutic agents, e.g., antisense oligonucleotides, small interfering RNAs, RNA aptamers, RNA-based vaccines, and mRNA drugs [3]. Generally, these functions are dependent on their three-dimensional (3D) structures, which can be determined by experimental methods like X-ray crystallography, nuclear magnetic resonance (NMR), or more recently cryo-electron microscopy (cryo-EM) [4]. However, the limited scope of known RNA structures obtained so far has led to an incomplete picture of the RNA structure in cells.

Fortunately, there are some computational methods that have been developed for predicting RNA 3D structures [4,5,6,7,8,9,10,11,12,13,14,15,16,17,18,19], among which the coarse-grained (CG) models have gained more attention [20,21,22,23,24,25,26,27,28,29]. For example, we have developed a three-bead CG model (using atoms of P, C4′, and N1 for pyrimidine or N9 for purine to represent each nucleotide) for RNA folding. Combining the sequence/salt-dependent CG potentials with Monte Carlo (MC)-simulated annealing or a replica exchange MC algorithm, the model can predict 3D structures and thermodynamic stability for RNA hairpins, duplexes, kissing complexes, and pseudoknots in monovalent/divalent ion solutions from sequences [29,30,31,32,33,34]. However, while the predicted CG structures from most of the CG models capture the primary topological information of RNA molecules, they are limited for practical applications due to the lack of atomistic details. Therefore, it is necessary to reconstruct the all-atomistic structures based on the CG structures.

Several RNA CG models have incorporated built-in all-atom reconstruction methods [21,23,24,25,28]. For instance, the five-bead CG model RNAJP (three for a base and two for a backbone) used a fragment replacement method to map the predicted CG structures to the corresponding all heavy-atom structures [25], that is, it first aligns the three CG beads in a base to the corresponding atoms in the standard A-form base, and the heavy atoms in the standard base are taken as the rebuilt heavy atoms. Then, to reconstruct the phosphate and sugar groups, backbone templates extracted from rRNAs are used to find the optimal superposition on three backbone atoms (i.e., P, C4′, and the next neighboring P) as well as a base atom. Subsequently, the reconstructed all-atom 3D structure is refined using the program QRNAs to fix the broken bonds and remove steric clashes [25,35]. Similarly, the rebuilding in SimRNA or HiRE-RNA is also performed using a built-in algorithm based on fragment matching and structural refinement [21,28]. Although these methods performed well in their respective models, the lack of standalone and user-friendly reconstruction programs makes their application in other CG models more challenging.

Compared to all-atom structure reconstruction programs for proteins [36,37,38], there are relatively few programs specifically designed for the CG structure reconstruction of RNA [39,40,41,42]. C2A (Coarse-to-Atomic), which is freely available at www.simtk.org/home/c2a (accessed on 25 January 2024), is a fully automated fragment-based method for reconstructing full atomic details from CG structures of RNAs using geometry knowledge from a reference database of one or more full atomic RNA crystal structures [40]. In C2A, one target RNA CG structure underwent segmentation into structural subsets (i.e., fragments such as helices, loops, and junctions) based on its secondary structure. Subsequently, CG matches for each fragment could be identified within a user-defined reference full atomic RNA 3D structure database (e.g., the *Thermus thermophilus* 16S ribosomal RNA), and these matches were then assembled using a Metropolis MC approach to generate a full atomic structure without significant atomic collisions. Finally, the reconstructed full atomic structure was minimized with molecular dynamics (MD) methods using the GROMACS software (https://www.gromacs.org/, accessed on 25 January 2024) [43] to eliminate any chemically unrealistic gaps or collisions. C2A was validated by seven RNA crystal structures, keeping only the C3′ position of each residue, with an average RMSD between reconstructed structures and the corresponding reference structures < 3.0 Å [40]. However, the method is limited by the quality of the template structure and information in the reference structure, and achieving convergence on a combination of fragments devoid of significant collisions could not be guaranteed.

On the contrary, NARall, a very simple tool (www.unres.pl, accessed on 25 January 2024) for reconstructing the full atomic structure of nucleic acids by sequentially restoring individual nucleotides from a CG model, relies minimally on known structural data (i.e., only needs nucleosides in standard A-RNA) and does not necessitate the provision of a secondary structure [41]. Nevertheless, the tool is specifically designed for the NARES-2P model (a CG model with two centers of interaction per repeating unit) [44] and lacks general applicability. Very recently, Perry et al. introduced Arena (https://github.com/pylelab/Arena, accessed on 25 January 2024), a highly accurate and user-friendly tool, capable of generating full atomic structures for any CG RNA model with a minimum of one atom per nucleotide [42]. Like NARall, Arena achieves reconstruction by superimposing A-form standard fragments onto a CG model, aligning with the positions of CG atoms. However, Arena distinguishes itself by overlaying entire nucleotides (>2 CG atoms) or fragments including adjacent/paired nucleotides (≤2 CG atoms) based on the number of CG atoms in each nucleotide, rather than individual nucleosides. Moreover, it refines bond lengths/angles, optimizes base/base-pair conformations, and eliminates clashing atom pairs through an iterative process, substituting the time-consuming MD or stochastic simulations utilized by other programs, which contributes to Arena’s faster computational speed. Benchmark testing with 361 experimental RNA structures [45] demonstrates that Arena attains superior accuracy (within 3.63 Å RMSD for a single P atom per nucleotide) and speed (e.g., 46× faster than C2A) when compared to other structure reconstruction programs [42]. However, both NARall and Arena exclusively utilize standard A-form nucleotide structural fragments as templates, overlooking the diversity in nucleotide configurations, especially in the loop regions.

Here, we present a very simple method named ABC2A to map a CG model to an all-atom structure by aligning several full atomic configurations with large differences in the corresponding CG atoms for each nucleotide. First, the template library including diversity nucleotide conformations was constructed by organizing experimental structure fragments based on their similarity to standard fragments. Second, ABC2A aligned all templates (with the same base type) to each CG nucleotide, and the best template was selected for replacement to achieve the full atomic structure reconstruction. Finally, simple structure refinement, including a bond check and clash elimination, was performed on the initial structure formed after traversing through all nucleotides, achieving rapid full structure reconstruction. ABC2A has been demonstrated to have exceptionally fast speed and high accuracy, with its source code written in C available at https://github.com/RNA-folding-lab/ABC2A, accessed on 25 January 2024.

## 2. Results

### 2.1. Overview of ABC2A

**Figure 1:**
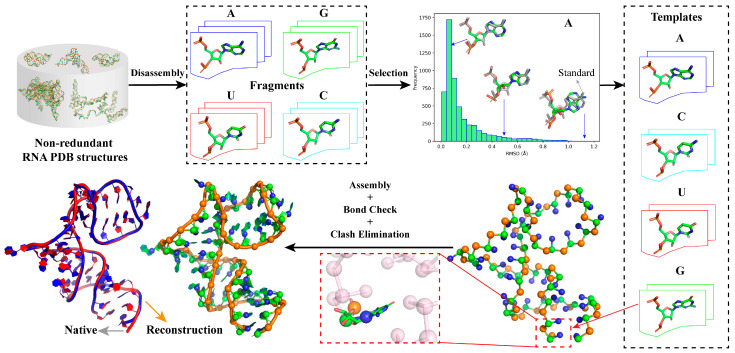
Overview of ABC2A. A full atomic nucleotide template library for each nucleotide type can be constructed by disassembling individual nucleotides from non-redundant RNA PDB structures and filtering out representative conformations according to their RMSDs to the corresponding standard A-form nucleotide structure. The full atomic reconstruction of an RNA CG model (e.g., three beads for one nucleotide) involves searching for the best match for each nucleotide’s CG atoms from the template library, followed by assembly, bond check, and clash elimination processes. All 3D structures or fragments are shown with PyMol (http://www.pymol.org, accessed on 25 January 2024).

As shown in Figure 1, the template libraries of four types of nucleotides (i.e., A, U, G, and C) in ABC2A were first constructed utilizing standard A-form nucleotide structures as well as full atomic structures of nucleotides with significant conformational differences disassembled from non-redundant PDB structures; see Materials and Methods. Then, for each nucleotide in a CG model (e.g., three beads for one nucleotide), all the templates in the library with the same base type were aligned to the nucleotide based on the CG atoms, and the best-matching one (with minimum RMSD) was selected to replace the CG beads, thus achieving the reconstruction of the full atomic structure. After sequentially traversing all nucleotides, the initial RNA full atomic structure was assembled, and it was further refined by a simple bond check and clash elimination process to avoid unrealistic gaps or collisions; see Figure 1. Although this method could apply to structures with different levels of coarse-graining, this work only discussed the feasibility and effectiveness of the simple approach using a three-bead CG model (see Materials and Methods) as an example.

### 2.2. Number of Fragments

The goal of ABC2A is to rapidly reconstruct the full atomic structure from a CG model using full atomic nucleotide fragments with diverse configurations in the PDB. To investigate the influence of the number of fragments used for reconstructing individual nucleotides on accuracy and time, we reconstructed full atomic structures from three-bead CG models using varying numbers of fragments (1–20) for the test set used by Perry et al., which includes 361 RNA single-stranded chains with lengths from 30 to 692 nt; see Materials and Methods or Refs. [42,45].

**Figure 2:**
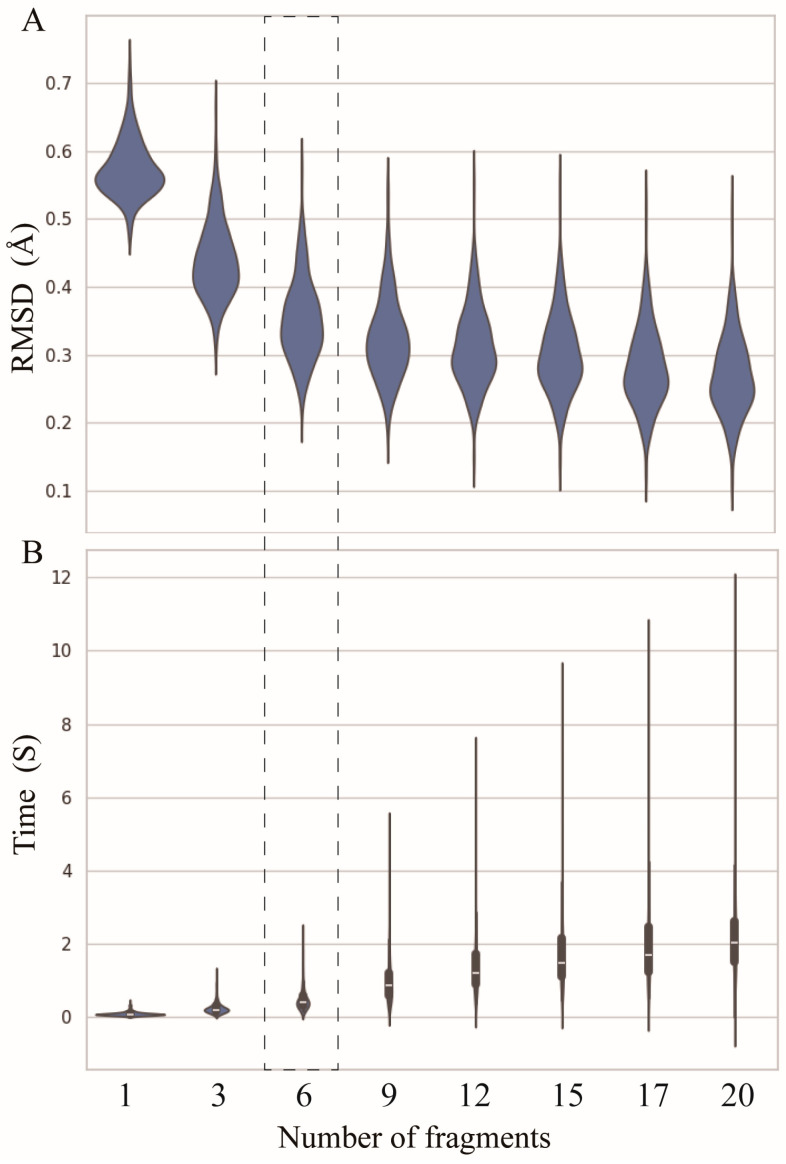
(**A**) The RMSDs between structures reconstructed by ABC2A and the experimental structures decrease with the number of fragments used in ABC2A. (**B**) The time required for reconstruction increases with the number of fragments. Six fragments were used in ABC2A (marked with a dashed box) for comparison with other methods.

As shown in Figure 2A, when only the standard A-form nucleotide fragments are used, the overall average RMSD between the reconstructed structures and the corresponding experimental structures is ~0.58 Å. The use of fragments derived from PDB structures significantly improved the reconstruction accuracy. For instance, when the number of fragments is equal to three (i.e., one standard fragment and two real fragments with different degrees of differences from the standard one), the overall mean RMSD decreases to ~0.44 Å, an improvement of ~0.14 Å.

Although the reconstruction accuracy increases with the increase in the number of fragments, the magnitude of the improvement becomes smaller, e.g., increasing the number of fragments from 9 to 12 results in only a 0.02 Å decrease in mean RMSD; see Figure 2A. However, as the number of fragments increases, the time required for full atomic structure reconstruction rapidly increases. When using only one fragment, the average time is less than 0.02 s (s), while with twenty fragments, the time increases to 2 s, which is 100 times slower than that of one fragment; see Figure 2B. It suggests that users can select different numbers of fragments within a certain range according to their needs, as selecting too many fragments is unnecessary. To compare to other programs, ABC2A utilized six fragments, where a good balance was achieved between accuracy and time (Figure 2).

### 2.3. Performance of ABC2A

**Figure 3:**
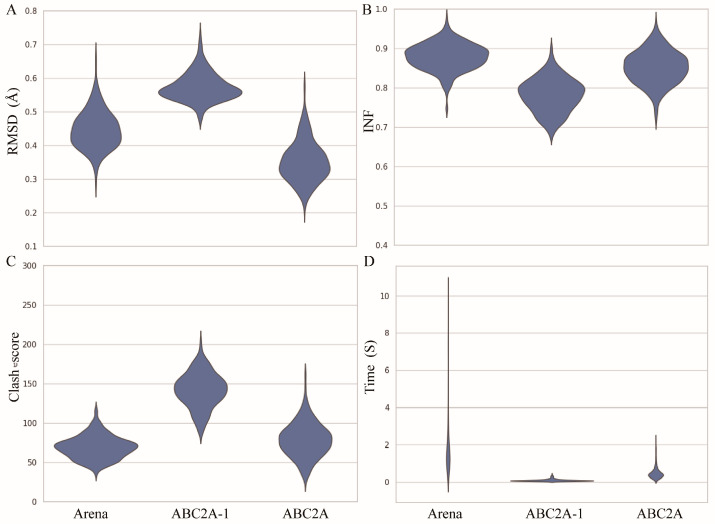
Performance of Arena, ABC2A-1 (using only one standard fragment for each nucleotide), and ABC2A (using six fragments for each nucleotide) on full atomic structure reconstruction from three-bead CG models for 361 RNAs in the test set, for which (**A**) RMSD, (**B**) INF, (**C**) clash score, and (**D**) runtime were calculated.

To further evaluate the reconstruction performance of ABC2A, we made comparisons with Arena on the test set across four metrics: RMSD, INF (interaction network fidelity), clash score, and runtime (see Materials and Methods) (Figure 3). Here, the INF is an evaluation of the reconstruction accuracy of different types of interactions, including Watson–Crick base-pairing, non-Watson–Crick base-pairing and base-stacking [46]. The range of INF values is from 0 to 1, where a higher value indicates that the interaction network of the reconstructed structure closely resembles that of the reference one. Since Arena outperformed existing methods (such as C2A [40], RCrane [39], PDBFixer in OpenMM [47], and Rosetta rna_thread [48]) in both speed and accuracy [42], we did not compare ABC2A with methods other than Arena in this paper. Figure 3 also shows the results from ABC2A using only the standard A-form fragment for each nucleotide (i.e., ABC2A-1).

For 361 RNA structures in the test set, the mean RMSD between structures reconstructed by ABC2A and experimental structures is ~0.34 Å, which is ~25% smaller than that of Arena (Figure 3A). Furthermore, there is no significant difference (*p*-value > 0.1) in the INF of ABC2A compared to Arena (Figure 3B). Although ABC2A contains ~8% more clashes than Arena (Figure 3C), it is ~5× faster than Arena (Figure 3D). Why is ABC2A so much faster? There are two main reasons: (1) It only needs to check and correct the bonds connecting two nucleotides, due to no change in bonds within assembled fragments; (2) During the clash elimination, ABC2A avoids altering bond lengths within fragments and repetitive iterations between nucleotides by simply rotating the orientation of the subsequent bases; see Materials and Methods.

Clearly, if only the standard fragments are used, the runtime is further reduced (~100× faster than Arena); however, the accuracy (e.g., ~24% higher in RMSD and ~10% lower in INF) and clash score of reconstructed structures become worse than Arena. This indicates that relying solely on standard fragments for structure reconstruction could neglect the diversity of nucleotide conformations, making it difficult to compensate for even through structure refinement.

**Figure 4:**
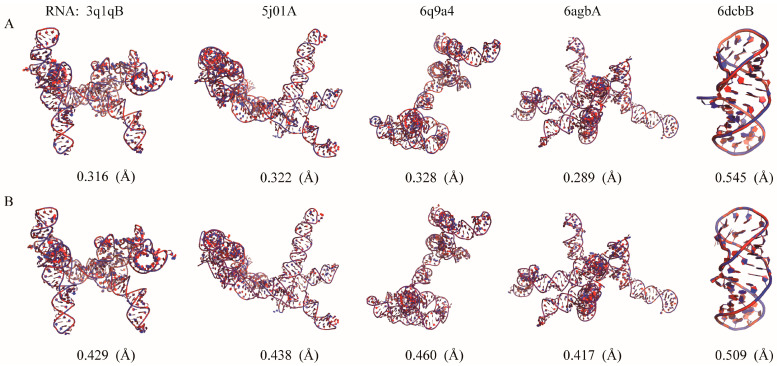
The native structures (red) of five RNAs are overlaid with the structures (blue) reconstructed by (**A**) ABC2A and (**B**) Arena from three-bead CG models. All 3D structures are shown with PyMol (http://www.pymol.org, accessed on 25 January 2024).

As shown in Figure 4, the structures reconstructed by Arena and ABC2A are both very close to the native structures (most of RMSD < 0.5 Å). It should be pointed out that for large RNAs with complex structures including lots of loops/single-stranded regions, ABC2A outperformed Arena. For example, for 369 nt of RNA in yeast ribonuclease P (PDB: 6agb), which includes five junction loops, seven hairpin loops, and a distinct pseudoknot [49], the RMSD between structures from ABC2A and PDB is 0.289 Å, which is 0.128 Å lower than that from Arena; see Figure 4. On the contrary, for a small pseudoknot (PDB: 6dcb; 37 nt), the reconstruction from Arena (0.509 Å) is slightly better than that from ABC2A (0.545 Å). This is understandable, as nucleotides in the stem closely resemble the standard A-form fragments, while those in loops exhibit diverse configurations and differences from the standard fragments. Therefore, ABC2A performs better when reconstructing complex structures with a significant number of loops, using a variety of fragments.

**Figure 5:**
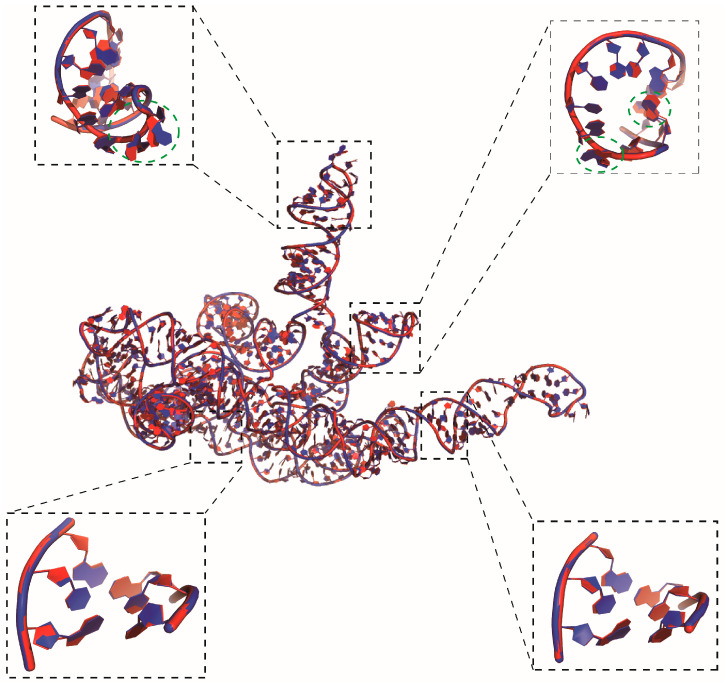
The native structure (red) of a 460 nt RNA overlaid with the reconstructed structure (blue) from ABC2A based on a three-bead model. Two regions in both the stem and loop are highlighted by boxes, where nucleotides in the reconstructed structure with significant deviations from the experimental structure are also marked with green dashed circles, to illustrate the performance of ABC2A. The 3D structures are shown with PyMol (http://www.pymol.org, accessed on 25 January 2024).

To ensure reconstruction speed, ABC2A utilized only six fragments that differ from the standard fragments. However, this could still be insufficient for reconstructing the diversity of loop regions. Figure 5 shows the reconstructed structure from ABC2A for a large RNA in an archaeal 30S initiation complex (PDB: 6swe; length: 460 nt) [50] with the RMSD of 0.428 Å deviated from the corresponding PDB structure. While the backbone of the reconstructed structure almost perfectly aligns with the experimental structure, there are still varying degrees of deviation, and even flips, in the orientation of the bases within the loop regions compared to the experimental one; see Figure 5, and this phenomenon is less common in the stem regions. This suggests that accurately reconstructing loop regions remains challenging when using A-form standard or limited fragments. Nonetheless, this work demonstrates that simply utilizing diverse structural fragments can effectively improve the accuracy of CG structure reconstruction, and the further optimization of representative fragment selection could further enhance the reconstruction performance of the method.

## 3. Discussion

To expedite and enhance the reconstruction of full atomic structures from CG models, we proposed a simple method, ABC2A, to build full atomic structures using six various fragments beyond one standard A-form fragment as a template for each nucleotide and validated its performance on a recent large dataset including 361 experimental RNAs. Comparison with the current state-of-the-art method Arena demonstrates that, based on a three-bead CG model, ABC2A achieves a faster and more accurate reconstruction of full atomic structures, with a shorter average runtime (~0.5 s vs. ~2.5 s) and a lower mean RMSD (~0.34 Å vs. ~0.45 Å). Moreover, the accuracy of ABC2A increases further with the use of more fragments for assembling each nucleotide, while its speed rapidly improves with fewer fragments utilized for assembly. The main reason why Arena and ABC2A are faster compared to existing methods is that they do not use stochastic simulations for structure refinement [42]. Additionally, compared with Arena, ABC2A further simplifies the clash elimination step to avoid repetitive iterations, resulting in even faster speed with only a minor sacrifice in clash score.

The focus of this study is to effectively enhance the accuracy of full atomic structure reconstruction utilizing simple alignment assembly by increasing the number of diverse fragments for each base type. Therefore, extensive testing was conducted only on the three-bead CG model. Although the method can be easily extended to any CG model, for models with fewer than three CG atoms per nucleotide, each fragment may involve multiple adjacent or paired nucleotides. In such cases, it might be challenging to select experimental fragments based on differences from the standard A-form fragments. Moreover, since diverse fragment configurations are generally from various loops or single-stranded regions, it is essential to further distinguish helix and non-helix fragments in known structures and then select representative fragments through clustering, respectively. In addition, since ABC2A does not rely on a secondary structure, it can be used to reconstruct disordered or unfolded RNA structures. However, for folded RNA structures, inferring the secondary structure from a CG model is generally not difficult, therefore the reconstruction speed and accuracy could be greatly improved by assembling helical and non-helical regions separately based on the secondary structure information.

## 4. Materials and Methods

### 4.1. The Three-Bead Coarse-Grained Model

The CG representation used here was the same as the CG model developed by us, which has been used to predict 3D structures and stability for RNAs in ion solutions [29,30,31,32,33,34]. In the model, an RNA is represented as a chain of nucleotides, where each nucleotide is reduced to three beads: the backbone phosphate bead (P) and sugar bead (C) coincide with the phosphate and C4′ atoms of a nucleotide, and the base beads (N) are placed at the base atoms linked to the sugar, that is, the N1 atom for pyrimidine or the N9 atom for purine; see Figure 1 and Ref. [29].

### 4.2. Construction of Nucleotide Template Library

**Figure 6:**
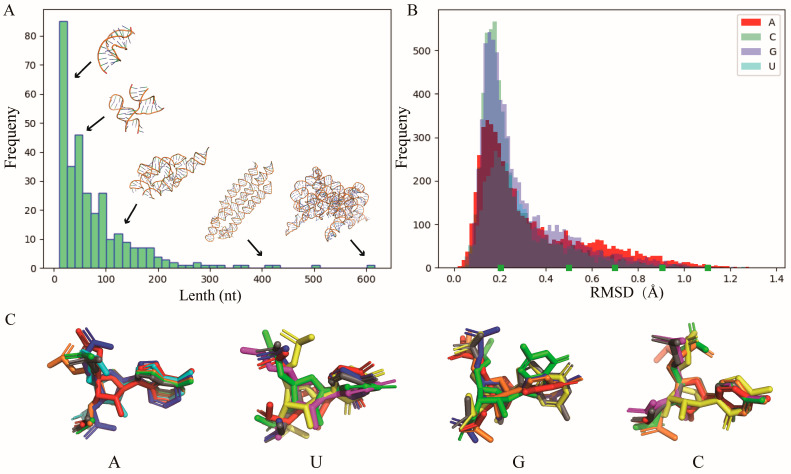
(**A**) The length distribution of 315 RNA PDB structures used in this work for nucleotide template library construction. The representative structures with different lengths are also shown inside. (**B**) The distributions of the RMSD between nucleotide fragments (A, U, G, and C) from PDB structures and the corresponding standard fragment from the A-form RNA structure. (**C**) The typical nucleotide conformations (colored) with different RMSDs (e.g., marks in **B**) superimposed on the standard structure (gray) for each type of base. All 3D structures are shown with PyMol (http://www.pymol.org, accessed on 25 January 2024).

Unlike existing methods such as Arena [42] and NARall [41], the full atomic templates of nucleotides utilized by ABC2A comprise not only standard A-form nucleotide fragments but also encompass diverse nucleotide fragments from PDB structures. The standard template for each nucleotide is from an ideal A-form RNA double helix generated by 3DNA [51]. To construct the template library, 1247 pure RNA structures with resolution < 3.5 Å were downloaded from the PDB (https://www.rcsb.org/, accessed on 25 January 2024), and then, the CD-HIT (http://www.bioinformatics.org/cd-hit/, accessed on 25 January 2024) was used to remove structures with sequence identity > 80%, resulting in 315 RNA structures; see Figure 6A for their length distribution. Subsequently, these RNA structures were segmented into individual nucleotide fragments (retaining the adjacent next P atom) and stored separately according to their base types (i.e., A, U, G, C).

Due to the large number of fragments for each base type (e.g., 5458 for A), it is challenging to use all of them. To obtain representative samples while preserving the diversity of fragments, we further calculated the RMSD between each fragment and its corresponding standard fragment. As shown in Figure 6B, most fragments exhibit similarity (e.g., with a RMSD < 0.3 Å) to their standard fragments, but there are still many fragments that deviate significantly from the standard fragments, which could contribute to the diversity of nucleotide configurations for each base type; see Figure 6C for the differences between typical conformations and their standard fragments. Finally, the standard structure along with conformations of varying similarity (the number of which can be user-defined) for each type of nucleotide can be used to construct a library, which serves as the template for the subsequent CG structure reconstruction.

### 4.3. Full Atomic Structure Assembly

The inputs of the ABC2A reconstruction program include the CG model in PDB format and the predefined template library (i.e., a series of nucleotide fragments in PDB format). Starting from the first nucleotide in the CG model including the base type and coordinates of CG beads, one full atomic template structure is retrieved from the library with the same base type. The center coordinates of CG beads and the corresponding atoms in the template are calculated, respectively, and both sets of centers are translated to the origin of the coordinate system. Then, the optimal rotation matrix is calculated using singular value decomposition (SVD) to achieve maximal overlap between the corresponding atoms in the template and the CG beads [52]. Meanwhile, the *RMSD* between the CG atoms of the two CG sets with the coordinates of *X* and *Y* can be calculated by $RMSD=\sqrt{\frac{1}{n}\sum_{i=1}^{n}{\left(X_{i}-Y_{i}\right)}^{2}}$, where *n* is the number of CG beads.

Subsequently, by traversing all templates (e.g., 6) in the library with the same base type, the template with the smallest RMSD is selected, and all its atoms are translated and rotated to replace the CG nucleotide. Finally, the coordinates of the replaced nucleotide atoms are translated back to the original position of the CG nucleotide, completing the full atomic reconstruction of the individual nucleotide. Following the above process, all nucleotides are sequentially reconstructed to obtain the initial full atomic structure.

### 4.4. Structure Refinement

Since each nucleotide is independently reconstructed in ABC2A, it is difficult to ensure the formation of covalent bonds between adjacent nucleotides (i.e., O3′-P). For the initial reconstructed full atomic structure, every inter-residue O3′-P bond is checked. If the bond length is larger than 1.8 Å or shorter than 1.4 Å (i.e., the deviation from the standard value of 1.6 Å is greater than 0.2 Å), the bond will be repaired by adjusting the two adjacent bond angles/lengths; see Ref. [42].

Furthermore, clashes between atoms are typically present in the reconstructed structure. As clashes between backbones (i.e., phosphate groups and sugar rings) are less likely to occur, to reduce computation, ABC2A only considers collisions related to the bases (i.e., phosphate group–base, sugar–base, and base–base). First, if the C4′–C4′ distance between two nucleotides is larger than 20 Å, it is not possible for the inter-atoms to clash. Otherwise, the distances between atoms within a given base and all atoms in the other nucleotide are calculated. If the distance between any two heavy atoms is smaller than the sum of the van der Waals radii of the two atoms, a clash is defined [42]. To avoid repetitive iterations, ABC2A minimizes clashes by adjusting the orientation of the base in the nucleotide with the larger sequence number. For instance, when the base atoms in the *j*-th nucleotide overlap with any atom in the *i*-th nucleotide (*j* > *i*), the entire base of the *j*-th nucleotide will rotate rigidly around the axis C4′-N1/N9, with N1 (purine) or N9 (pyrimidine) as the center, by a small angle (e.g., π/20) based on the direction vector between the colliding atoms. The *i*-th nucleotide remains stationary. This process is repeated until the *j*-th base no longer overlaps with the atoms in the *i*-th nucleotide or until a predefined number of steps (e.g., 100) is reached.

### 4.5. Test Sets and Performance Evaluation

To test the ABC2A, the dataset of 361 non-redundant RNA structures (with sequence identity < 80%) curated from the PDB, which was recently reported by Perry et al. [42], was used. These structures are single chains (30–692 nt) with more than 10 canonical base pairs from RNAs including rRNAs, tRNAs, snRNAs, introns, and riboswitches [42,45]. For each structure in the dataset, the target CG model for reconstruction was generated by retaining the information of corresponding CG atoms and removing the nucleotides with incomplete CG atoms.

The accuracy of the reconstruction was evaluated by RNA puzzles metrics such as the RMSD, clash score, and INF [46,53]. The RMSD between the reconstructed structure and the corresponding experimental PDB structure was calculated by TMscore (https://zhanggroup.org/TM-score/, accessed on 25 January 2024) [54,55]. The INF, which is defined as the Matthews correlation coefficient between the base-pairing/stacking interactions of the reference structure and that of the reconstructed structure (1 indicating perfect consistency), was calculated by RNA_assessment (https://github.com/RNA-Puzzles/RNA_assessment, accessed on 25 January 2024) [56]. The clash score that reports serious steric clashes identified in an RNA 3D structure was calculated by MolProbity (http://molprobity.biochem.duke.edu/, accessed on 25 January 2024) [57].

## Data Availability

The data presented in this study are available in article.
